# Survival from cancer of the stomach in England and Wales up to 2001

**DOI:** 10.1038/sj.bjc.6604574

**Published:** 2008-09-23

**Authors:** E Mitry, B Rachet, M J Quinn, N Cooper, M P Coleman

**Affiliations:** 1 Département d'Hépatogastroentérologie et Oncologie Digestive, Centre Hospitalo-Universitaire Ambroise-Paré, 9 avenue Charles de Gaulle, F-92100 Boulogne, France; 2 Cancer Research UK Cancer Survival Group, Non-Communicable Disease Epidemiology Unit, Department of Epidemiology and Population Health, London School of Hygiene and Tropical Medicine, Keppel Street, London WC1E 7HT, UK; 3 Social and Health Analysis and Reporting Division, Office for National Statistics (Room FG/114), 1 Myddelton Street, London EC1R 1UW, UK

The steady, long-term decline in stomach cancer incidence and mortality has seen its relative frequency in England and Wales fall from 5% of all malignant neoplasms in the 1970s to 2.3% by 2002 (Coleman *et al*, 1993; Cooper *et al*, 2005). It is now only the sixth most common cancer in men and the tenth most common in women. The decline in incidence is probably related to changes in diet and nutrition, improved preservation of food and a reduction in the prevalence of *Helicobacter pylori* infection and in tobacco smoking (Parkin, 2001). Incidence is higher in men (sex ratio about 2.5 to 1), in the north of England, and in more deprived socioeconomic groups (Coleman *et al*, 1999). In the late 1990s, incidence was still 1.35 times higher in the most deprived socioeconomic group than in the most affluent, but this ratio was lower than in the early 1990s (1.41), because incidence fell by about 9% in the most deprived group over this decade, slightly faster than in the most affluent groups (Figure 1).

We analysed data for 112 367 adults registered with cancer of the stomach in England and Wales during the period 1986–1999, approximately 81% of the 139 154 eligible for analysis. The vital status for 1.6% of eligible patients was unknown on 5 November 2002, and they were excluded from analysis. Most of the other exclusions were for a recorded survival time of zero (date of diagnosis same as date of death; 14.2% of cases): some of these patients did die on the day of diagnosis, but most were registered solely from a death certificate, and their survival time was unknown. The two groups could not be distinguished in these data. As such patients may have shorter-than-average survival (Berrino *et al*, 1995), the potential impact on trends and inequalities in survival needs to be considered. The percentage of eligible patients excluded with zero recorded survival fell from 14% for those registered in 1990 to 9% for 1999, but this trend was similar in all deprivation groups, and any impact on trends in the deprivation gap in survival is likely to have been small. Patients for whom stomach cancer was not the first primary malignancy (3%) were also excluded.

The proportion of stomach cancers specified as arising in the cardia rose from 18% during 1991–1994 (ICD-9 151.0) to 24% during 1995–1999 (ICD-10 C16.0). This shift in the sub-site distribution appears more likely to be real than an artefact due to more specific pathological reporting, even though the proportion with unspecified sub-site fell from 60 to 52%. This is because the proportion of gastric tumours arising in the cardia has been rising steadily from the early 1970s (6%) to the late 1980s (15%) (Coleman *et al*, 1999). The proportion of cancers specified as adenocarcinoma rose from 60 to 72% during the 1990s in these data, and an increase in the incidence of adenocarcinomas at the oesophagogastric junction has been reported several times (Powell and McConkey, 1990; Newnham *et al*, 2003). The proportion of cancers at other sub-sites of the stomach was stable during the 1990s: fundus (1.5%), body (3%), antrum (6%), pylorus (2%), lesser curvature (6–7%) and greater curvature (2–3%). The shift towards more cardiac tumours may affect overall survival, because proximal tumours of the stomach tend to be more difficult to remove.

## Survival trends

**Figure 2:**
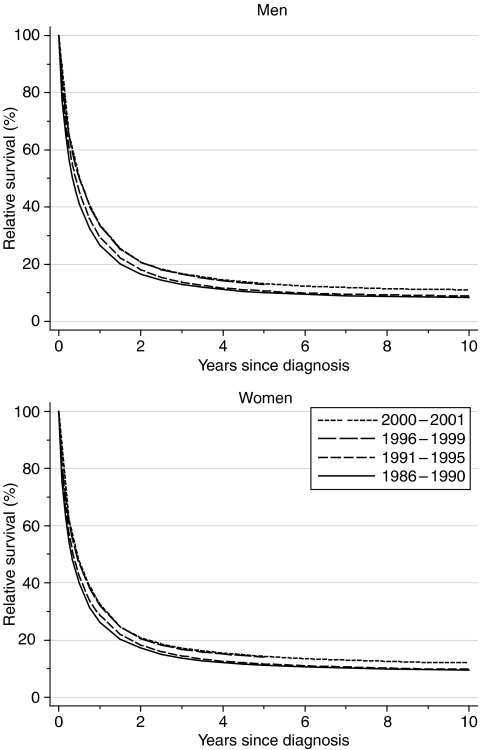
Relative survival (%) up to 10 years after diagnosis by sex and calendar period of diagnosis: England and Wales, adults (15–99 years) diagnosed during 1986–1999 and followed up to 2001. Survival estimated with cohort or complete approach (1986–1990, 1991–1995, 1996–1999) or hybrid approach (2000–2001) (see Rachet *et al*, 2008).

Survival has been increasing steadily and significantly. One-year survival in men rose from 26.6% for those diagnosed during 1986–1999 to 33.5% for those diagnosed during 1996–1999 (Figure 2). The fitted, deprivation-adjusted average increase of 4.7% every 5 years was statistically significant (Table 1). The increase was similar in women, from 26.3 to 32.1% (+4.2% every 5 years). The increase in 5-year survival was less marked, but still statistically significant: it rose to 12.9% for men diagnosed at the end of the 1990 s (+2.0% every 5 years) and 14.0% for women (+1.7% every 5 years). Ten-year survival for those diagnosed during 1991–1995 was 9–10%, not much less than the 5-year survival rate, suggesting that most of the excess mortality in stomach cancer patients occurs in the first 5 years.

Short-term predictions of survival for patients diagnosed during 2000–2001, using hybrid analysis (Brenner and Rachet, 2004), suggest that 1-year survival will remain around 33–34%, with a small continuing increase to around 11–14% in survival at 5 or more years after diagnosis (Table 1).

## Deprivation

**Figure 3:**
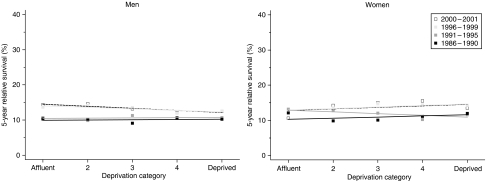
Trends in the deprivation gap in 5-year relative survival (%) by sex and calendar period of diagnosis: England and Wales, adults (15–99 years) diagnosed during 1986–1999 and followed up to 2001.

For men, short-term survival is significantly worse among the most deprived groups (Table 2, Figure 3). The deprivation gap widened from −2.1% for those diagnosed during 1986–1990 to −4.8% for those diagnosed during 1996–1999, although the average widening of the gap (−1.3% every 5 years) was not itself statistically significant. The deprivation gap in 5-year survival was less marked. Hybrid analysis suggests that the deprivation gap in 1-year survival for men could increase to around 8%.

No clear trend in the deprivation gap was seen for women.

## Comment

The significant improvement in stomach cancer survival in England and Wales since 1990 continues the trend observed since the early 1970s. One-year survival improved more rapidly in the 1990s (4–5% every 5 years) than in the 1970s and 1980s (2% every 5 years), whereas improvements in longer-term survival have slowed down. Gains in 5-year survival for patients diagnosed in the 1990s (2% every 5 years) were notably smaller than in the two previous decades (4% every 5 years) (Coleman *et al*, 1999). This pattern probably reflects recent improvements in perioperative mortality (Berrino *et al*, 1999; Msika *et al*, 2000). The widening of the deprivation gap in men could suggest that men in the most deprived group have not benefited from this reduction in operative mortality.

## Figures and Tables

**Figure 1:**
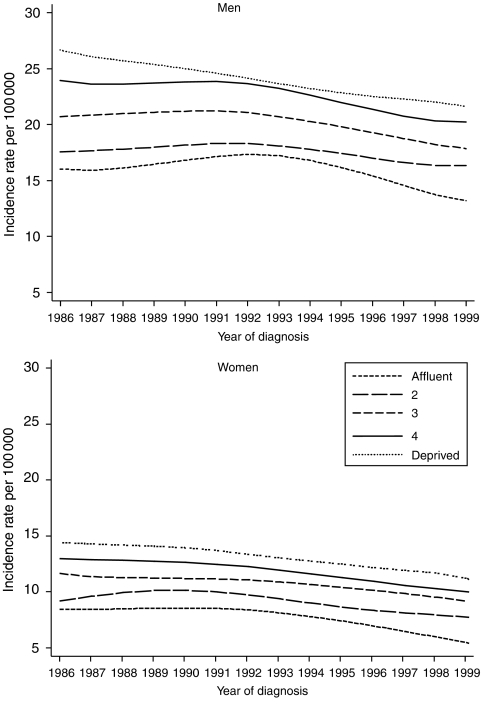
Trends in the age-standardised incidence of stomach cancer in adults aged 15–99 years, by sex and deprivation group: England and Wales, 1986–1999.

**Table 1:** Trends in relative survival (%) by sex, time since diagnosis and calendar period of diagnosis: England and Wales, adults (15–99 years) diagnosed during 1986–1999 and followed up to 2001

		**Calendar period of diagnosis^a^**						**Average change (%)**		**Prediction^c^ for patients**	
		**1986–1990**		**1991–1995**		**1996–1999**		**every 5 years^b^**		**diagnosed during 2000–2001**	
**Time since diagnosis**		**Survival (%)**	**95% CI**	**Survival (%)**	**95% CI**	**Survival (%)**	**95% CI**	**Survival (%)**	**95% CI**	**Survival (%)**	**95% CI**
1 year	Men	**26.6**	(26.1, 27.2)	**29.5**	(28.9, 30.1)	**33.5**	(32.8, 34.1)	**4.7** ^**^	(3.3, 6.0)	**33.8**	(32.8, 34.8)
	Women	**26.3**	(25.6, 27.0)	**28.6**	(27.9, 29.4)	**32.1**	(31.2, 33.0)	**4.2** ^**^	(2.4, 5.9)	**32.6**	(31.3, 34.0)
5 years	Men	**10.1**	(9.7, 10.5)	**10.6**	(10.2, 11.1)	**12.9**	(12.3, 13.5)	**2.0** ^**^	(0.9, 3.1)	**13.2**	(12.5, 14.0)
	Women	**11.1**	(10.6, 11.7)	**11.6**	(11.1, 12.2)	**14.0**	(13.2, 14.9)	**1.7** ^*^	(0.3, 3.2)	**14.3**	(13.2, 15.4)
10 years	Men	**8.4**	(7.9, 8.8)	**8.9**	(8.4, 9.4)			**1.0**	(−0.7, 2.8)	**11.0**	(10.2, 11.9)
	Women	**9.4**	(8.8, 10.0)	**9.9**	(9.2, 10.6)			**3.2** ^*^	(0.7, 5.7)	**12.1**	(11.0, 13.2)

CI=confidence interval.

a Survival estimated with cohort or complete approach (see Rachet *et al*, 2008).

b Mean absolute change (%) in survival every 5 years, adjusted for deprivation (see Rachet *et al*, 2008).

c Survival estimated with hybrid approach (see Rachet *et al*, 2008).

^*^P<0.05; ^**^P<0.01.

**Table 2:** Trends in the deprivation gap in relative survival (%) by sex, time since diagnosis and calendar period of diagnosis: England and Wales, adults (15–99 years) diagnosed during 1986–1999 and followed up to 2001

		**Calendar period of diagnosis^a^**						**Average change (%)**		**Prediction^c^ for patients**	
		**1986–1990**		**1991–1995**		**1996–1999**		**every 5 years^b^**		**diagnosed during 2000–2001**	
**Time since diagnosis**		**Deprivation gap (%)**	**95% CI**	**Deprivation gap (%)**	**95% CI**	**Deprivation gap (%)**	**95% CI**	**Deprivation gap (%)**	**95% CI**	**Deprivation gap (%)**	**95% CI**
1 year	Men	−2.1*	(−3.8, −0.5)	−2.2*	(−3.9, −0.5)	−4.8**	(−6.9, −2.8)	**−1.3**	(−2.6, 0.1)	−7.9**	(−10.8, −4.9)
	Women	**0.4**	(−1.8, 2.5)	−3.0*	(−5.3, −0.7)	**−1.6**	(−4.4, 1.2)	**−1.3**	(−3.1, 0.5)	**−1.7**	(−5.8, 2.3)
5 years	Men	**0.3**	(−0.9, 1.6)	**0.3**	(−0.9, 1.6)	**−1.7**	(−3.6, 0.1)	**−0.8**	(−1.9, 0.3)	−2.3*	(−4.7, 0.0)
	Women	**1.3**	(−0.4, 2.9)	−2.0*	(−3.8, −0.2)	**1.7**	(−0.9, 4.3)	**−0.5**	(−2.0, 1.1)	**1.7**	(−1.3, 4.7)
10 years	Men	**0.8**	(−0.5, 2.0)	**0.1**	(−1.3, 1.5)			**−0.7**	(−2.6, 1.2)	**−2.0**	(−4.5, 0.5)
	Women	**1.4**	(−0.4, 3.1)	**−1.8**	(−3.9, 0.3)			−3.2*	(−5.8, −0.5)	**0.8**	(−2.3, 4.0)

CI=confidence interval.

a Survival estimated with cohort or complete approach (see Rachet *et al*, 2008).

b Mean absolute change (%) in the deprivation gap in survival every 5 years, adjusted for the underlying trend in survival (see Rachet *et al*, 2008).

c Survival estimated with hybrid approach (see Rachet *et al*, 2008).

^*^P<0.05; ^**^P< 0.01.
